# Assessment of FDA-Approved Drugs as a Therapeutic Approach for Niemann-Pick Disease Type C1 Using Patient-Specific iPSC-Based Model Systems

**DOI:** 10.3390/cells11030319

**Published:** 2022-01-18

**Authors:** Christin Völkner, Supansa Pantoom, Maik Liedtke, Jan Lukas, Andreas Hermann, Moritz J. Frech

**Affiliations:** 1 Translational Neurodegeneration Section “Albrecht Kossel”, Department of Neurology, University Medical Center Rostock, 18147 Rostock, Germany; Christin.Voelkner@med.uni-rostock.de (C.V.); supansapantoom@yahoo.com (S.P.); Maik.Liedtke@med.uni-rostock.de (M.L.); jan.lukas@med.uni-rostock.de (J.L.); andreas.hermann@med.uni-rostock.de (A.H.); 2 Center for Transdisciplinary Neurosciences Rostock (CTNR), University Medical Center Rostock, 18147 Rostock, Germany; 3 Deutsches Zentrum für Neurodegenerative Erkrankungen (DZNE) Rostock/Greifswald, 18147 Rostock, Germany

**Keywords:** pharmacological chaperones, NPC1, in silico screening, molecular docking, induced pluripotent stem cells

## Abstract

Niemann-Pick type C1 (NP-C1) is a fatal, progressive neurodegenerative disease caused by mutations in the *NPC1* gene. Mutations of *NPC1* can result in a misfolded protein that is subsequently marked for proteasomal degradation. Such loss-of-function mutations lead to cholesterol accumulation in late endosomes and lysosomes. Pharmacological chaperones (PCs) are described to protect misfolded proteins from proteasomal degradation and are being discussed as a treatment strategy for NP-C1. Here, we used a combinatorial approach of high-throughput in silico screening of FDA-approved drugs and in vitro biochemical assays to identify potential PCs. The effects of the hit compounds identified by molecular docking were compared in vitro with 25-hydroxycholesterol (25-HC), which is known to act as a PC for NP-C1. We analyzed cholesterol accumulation, NPC1 protein content, and lysosomal localization in patient-specific fibroblasts, as well as in neural differentiated and hepatocyte-like cells derived from patient-specific induced pluripotent stem cells (iPSCs). One compound, namely abiraterone acetate, showed comparable results to 25-HC and restored NPC1 protein level, corrected the intracellular localization of NPC1, and consequently decreased cholesterol accumulation in NPC1-mutated fibroblasts and iPSC-derived neural differentiated and hepatocyte-like cells. The discovered PC altered not only the pathophysiological phenotype of cells carrying the I1061T mutation— known to be responsive to treatment with PCs—but an effect was also observed in cells carrying other NPC1 missense mutations. Therefore, we hypothesize that the PCs studied here may serve as an effective treatment strategy for a large group of NP-C1 patients.

## 1. Introduction

Niemann-Pick type C (NP-C) is a neurodegenerative lysosomal storage disorder (LSD), inherited in an autosomal recessive manner [1]. Mutations in either of the two genes, *NPC1* or *NPC2* (with *NPC1* accounting for 95% of cases), are causative for this disease for which there is no curative treatment [2]. Both genes encode for intracellular cholesterol transporter proteins: NPC1, a lysosomal transmembrane protein, and NPC2, a soluble lysosomal protein. Thus, mutations in both genes lead to an accumulation of cholesterol and sphingolipids, depicting the hallmark of the disease.

Even though the primary genetic defect of Niemann-Pick type C1 (NP-C1) has already been known since 1997 [3], this has not led to an FDA-approved treatment for the disease yet. To date, more than 500 disease-causing mutations for NP-C1 are listed in the Human Gene Mutation Database [4]. Most of the mutations are missense mutations [4] that lead to amino acid substitutions. They often result in the inability of the respective protein to properly fold into its native conformation, which is characterized by the most energetically favorable state [5]. Subsequently, incorrectly folded proteins are recognized by the endoplasmic reticulum (ER) quality control system, retro-translocated into the cytosol and degraded by the ER-associated degradation (ERAD) machinery [5]. In the context of NP-C1, this is particularly true for the prevalent I1061T mutation, found in 20% of patients of western European descent, especially France and the UK [6,7]. As shown by Gelsthorpe and colleagues, this missense mutation does not lead to loss of function per se but shows a folding defect, with the misfolded protein being retained in the ER [8] and targeted for proteasomal degradation in the cytosol. Consequently, the NPC1 protein is not transported to the lysosomal membrane and cannot fulfill its function, namely, cholesterol transport. The small proportion of NPC1^I1061T^, which escapes ERAD and reaches its final destination in the lysosomal membrane, had been shown to be functional [8]. This argues that strategies to support proper folding and trafficking of NPC1 protein may allow the recovery of the I1061T mutant. For this purpose, so-called pharmacological chaperones can be used to protect the mutant protein from degradation and restore the correct localization of misfolded proteins. 

The term pharmacological chaperones was first used by Morello and co-workers in 2000 [9]. The underlying mechanism of pharmacological chaperones is based on the concept of substances that specifically bind to the folding intermediate of the misfolded protein, assisting the correct folding and preventing their recognition by the quality control system. The result of PC treatment is an increased amount of mature protein, improved trafficking of the protein towards its final destination, the lysosome, and partial restoration of the functionality [5]. Due to the small size of PCs, they are likely to cross the blood–brain barrier and, therefore, have a potential for the treatment of diseases involving the central nervous system, such as NP-C1 [5]. In the context of NP-C1, an ideal chaperone shows a good affinity with the NPC1 protein in the ER to assist folding, but a good dissociation from the NPC1 protein in the lysosome, which should be favored by the acidic environment, to evacuate the cholesterol-binding pocket and recover the transporting function. Several LSDs have been treated with pharmacological chaperones, such as Fabry disease with 1-Deoxygalactonojirimycin (DGJ, Migalastat) [10]. However, until now, no pharmacological chaperone has been approved for NP-C1. 

To enable a rapid translation from research to clinics, there is a need for a sensitive high-throughput assay to screen for potential pharmacological chaperones with high affinity to NPC1. Therefore, we used a combinatorial approach involving high-throughput molecular docking and cell-based in vitro assays to identify potential pharmacological chaperones for misfolded NPC1 proteins. To support the translation of identified PCs into potential clinical use, we used FDA-approved drugs of the DrugBank [11,12] for molecular docking. In the next step, we tested the identified hit compounds in the widely used cell model of fibroblasts derived from NP-C1 patients. Finally, we extended our study to iPSC-derived neural differentiated and hepatocyte-like cells and performed the in vitro assays in these cell types, which are severely affected by NP-C1 disease.

## 2. Materials and Methods

### 2.1. Molecular Docking

Molecular docking was performed using the open-source software Autodock Vina version 1.1.2 [13]. The software was implemented on the high-performance Linux cluster (HPC) of Rostock University. Performing the docking simulation on the HPC server allowed us to accomplish the high throughput screening of large compound databases with a docking efficiency of 8 s per ligand. A total of 2162 compound structures from the DrugBank (DB) [12] database were filtered to remove compounds with high complexity and large structure, as well as compounds that contain toxic moieties, using a server called FAF-Drugs4 [14]. After the initial filtration, 1920 compounds were obtained. These compound structures were then optimized using energy minimization approach to get well-defined 3D structures. The structural format of the ligands was conversed to Autodock format called PDBQT file using the ligand preparation script called prepare_ligand4.py. During the process, the Gasteiger charges and polar hydrogen were added and the rotable bonds were defined. The X-ray crystallographic structure of the N-terminal domain (NTD) of NPC1 in complex with cholesterol was used as the receptor to obtain an open conformation of the cholesterol-binding site as the docking site. The structure was retrieved from Protein Data Bank (PDB) (PDB ID: 3GKI). The structure of the receptor was prepared for docking by removing the cholesterol structure, water, and ion molecules. Moreover, the structure was checked in the COOT software to ensure that the structure had no residue with missing atoms and with an alternative conformation. The PDBQT file of the receptor was prepared manually on the Autodock tool, and the charges and polar hydrogen were added to the receptor structure. The input parameters of the docking, including the size and coordinate of the grid box, receptor, and exhaustiveness value were indicated in the submission script. The docking grid box had the dimensions of 12.75 Å × 12.25 Å × 20.25 Å and the coordinates that center at the position of the cholesterol were used as the docking area. The exhaustiveness value was defined at 8.

### 2.2. Reagents

Chemical reagents used in this study and their commercial sources were as follows: dydrogesterone (HY-B0257A), lumacaftor (HY-13262), abiraterone acetate (HY-75054), eltrombopag (HY-15306), ethinylestradiol (HY-B0216), and nafamostat (HY-B0190) were from MedChemExpress (Monmouth Junction, NJ, USA). Methyltestosterone (M1800000), quinestrol (E7887), nandrolone phenpropionate (BP260), and testosterone propionate (T1875) were from MERCK (Darmstadt, Germany). 25-Hydroxycholesterol (sc-214091) was from Santa Cruz Biotechnology (Dallas, TX, USA). Compounds were solubilized in DMSO and used at a concentration of 10 µM for 48 h. The proteasome inhibitor MG132 (Thermo Fisher Scientific, Waltham, MA, USA) was used at 10 µM for 24 h.

### 2.3. Cell Culture and Drug Treatment

Human dermal fibroblast cell lines were obtained from the NIGMS Human Genetic Cell Repository at the Coriell Institute for Medical Research, Camden, NJ, USA (GM08398 (control), homozygous NPC1^I1061T/I1061T^ (GM18453), and compound heterozygous NPC1^E612D/P543Rfs*20^ (GM18436)), or Centogene AG, Rostock, Germany (homozygous NPC1^Y394H/Y394H^ (A113011)), respectively. Cells were cultured in Dulbecco’s modified Eagle medium containing high glucose supplemented with 10% fetal bovine serum and 1% penicillin–streptomycin (all Thermo Fisher Scientific, Waltham, MA, USA). Cells were passaged once a week and seeded at a density of 30,000 cells/cm^2^. To seed fibroblast on glass coverslips, they were coated with 0.1% gelatin (Sigma-Aldrich, St. Louis, MO, USA) in water for 30 min at 37 °C. Cells were allowed to adhere for 24 h. The next day, cells were incubated with compounds for 48 h at 37 °C and 5% CO_2_. DMSO solvent controls (0.1%) were run in parallel to the treatments with the compounds. 

### 2.4. hiPSC Cultivation and Neural and Hepatic Differentiation

Induced pluripotent stem cells were cultured on Matrigel (Corning, Corning, NY, USA) in mTeSR1 medium and passaged every 5 days using ReleSR (both Stemcell Technologies, Köln, Germany). Differentiation of iPSCs into neural differentiated or hepatocyte-like cells was carried out following published protocols [15].

### 2.5. Filipin Staining and Fluorescence Microscopy

For filipin staining, the cells were grown on glass coverslips. To compensate for the lack of FBS during culture of neural differentiated and hepatocyte-like cells, 60 µg/mL LDL (Sigma-Aldrich, St. Louis, MO, USA) were added to the culture medium for 48 h as an external cholesterol source prior to fixation. Cells were fixed with 4% PFA in PBS at pH 7.4 for 10 min at room temperature and stained with 0.1 mg/mL filipin (Polysciences Europe GmbH, Hirschberg an der Bergstraße, Germany) for 45 min in the dark. After washing with PBS, the coverslips were mounted with Fluoromount-G^®^ (SouthernBiotech, Birmingham, AL, USA). Microscopy was performed using either a Laser Scanning Microscope 900 (LSM 900, Zeiss, Oberkochen, Germany) or a Biozero8000 microscope system (Keyence, Osaka, Japan). The LSM 900 was equipped with a motorized scanning stage 130 × 100 STEP, a laser module URGB with a 405 nm diode laser, a Plan-APOCHROMAT 63×/1.4 Oil objective and a GaAsP-PMT detector; images were taken using the ZEN imaging software version 3.1 (all Zeiss, Oberkochen, Germany). On the Biozero8000 microscope system, images were captured using the following settings: 20×/NA0.75 objective, DAPI-B filter cube (EX, 360/40; DM, 400; and BA, 460/50), exposure time for DAPI (filipin) 1/15 s. Quantitative analysis of filipin images from 10 randomly chosen fields of cells/experiment was performed using ImageJ software version 1.8.0_172 (NIH, Bethesda, MD, USA) based on the ‘Lysosome-like storage organelle (LSO) compartment ratio assay’ [16]. For the calculation of the LSO ratio, two different thresholds were applied, the lower one defining the total area of the cells, the higher one to identify the bright, filipin-stained perinuclear LSO regions of the cells. The ‘LSO compartment ratio’ is defined as the total fluorescence intensity of the filipin staining above the high threshold divided by the number of pixels above the low threshold.

### 2.6. Colocalization Analysis

Fibroblasts, neural differentiated, and hepatocyte-like cells were grown on coverslips and washed with phosphate-buffered saline (PBS) with calcium and magnesium (PBS^+/+^) prior to fixation. Cells were fixed with ice-cold acetone for 5 min, subsequently washed with PBS^+/+^, and incubated with permeabilization buffer (0.1% Triton X-100 in PBS) for 5 min on ice. After another washing step with PBS^+/+^, cells were blocked with 1% BSA/PBS for 30 min at room temperature, followed by incubation with primary antibodies in 1% BSA/PBS at 4 °C overnight. Antibodies used were rabbit anti-NPC1 (1:1000, Abcam, Cambridge, UK) and mouse anti-LAMP2 (1:50, Abcam, Cambridge, UK). Next, cells were washed with 1% BSA/PBS, followed by incubation with anti-rabbit IgG conjugated with Alexa488 and anti-mouse IgG conjugated with Alexa568 secondary antibody (Invitrogen, Waltham, MA, USA) diluted 1:1000 for 2 h at room temperature. Finally, cells were stained with DAPI (5 min, 250 ng/mL) and coverslips were mounted using Fluoromount-G^®^ (SouthernBiotech, Birmingham, AL, USA). Microscopy was performed using a Laser Scanning Microscope 900 equipped with a motorized scanning stage 130 × 100 STEP, a laser module URGB with 405 nm, 488 nm and 561 nm diode laser, a Plan-APOCHROMAT 63×/1.4 Oil objective, and a GaAsP-PMT detector, using the ZEN imaging software (all Zeiss, Oberkochen, Germany). Colocalization analysis was performed using ImageJ software (NIH, Bethesda, MD, USA) and the plugin JaCoP (Just another Colocalization Plugin) to determine Pearson’s correlation coefficient.

### 2.7. Western Blot Analysis

Harvest of cells for Western blot analysis was carried out by lysis in RIPA buffer (20 mM TRIS, 137 mM NaCl, 12 mM sodium deoxycholate, 2 mM EDTA, 0.1% SDS, 1% Triton X-100, and 10% Glycerol in water) containing complete protease inhibitor cocktail (Roche Diagnostics, Rotkreuz, CH) for 25 min on ice. Insoluble cell debris was pelleted (15,000× *g* at 4 °C, 25 min), followed by determination of the total protein concentration in the supernatant using the Pierce BCA Protein Assay Kit (Thermo Fisher Scientific, Waltham, MA, USA). For Western blotting, 20 µg of non-boiled protein samples (37 °C, 5 min) were mixed with 5× Laemmli buffer (125 mM TRIS, 20% glycerol, 2% SDS, 5% β-mercaptoethanol, 10% bromphenol blue in water), separated by SDS-PAGE with Criterion™ TGX precast polyacrylamide gels (4–15%, Bio-Rad Laboratories GmbH, Feldkirchen, Germany), and run for 5 min at 100 V, followed by 20 min at 300 V. Precision Plus Protein Dual Xtra Standards (Bio-Rad Laboratories GmbH, Feldkirchen, Germany) was used as a molecular weight marker. Proteins were transferred to a nitrocellulose membrane on a semidry transfer apparatus (Trans-Blot Turbo) (both Bio-Rad Laboratories GmbH, Feldkirchen, Germany). Membranes were blocked for 1 h at room temperature with 5% skim dry milk (Sigma-Aldrich, St. Louis, MO, USA) in TBS-Tween 0.1% (TBS-T). Membranes were incubated with rabbit anti-NPC1 antibody (1:500, Abcam, Cambridge, UK) and mouse anti-beta-actin (1:10.000, Abcam, Cambridge, UK) in 3% skim dry milk in TBS-Tween 0.1% (TBS-T) overnight at 4 °C. Next, membranes were washed with TBS-T and incubated with fluorescence-coupled secondary antibodies (goat anti-rabbit IgG (H&L) Antibody DyLight™ 680 and goat anti-mouse IgG (H&L) Antibody DyLight™ 800, both Rockland Immunochemical, Inc., Limerick, PA, USA), 1:10.000 in 3% nonfat dry milk in TBS-T, for 2 h at room temperature. Finally, membranes were washed 3× with TBS-T and 1× with TBS and dried prior to analysis. Bands were detected and visualized using the Odyssey Infrared Imaging System (LI-COR Biosciences GmbH, Bad Homburg vor der Höhe, Germany). Densitometry of the resulting bands was performed in ImageStudioLite. The signals were normalized to those obtained for beta-actin. Relative levels of NPC1 protein resulting from treatment were compared to the corresponding DMSO treatment. As the used anti-NPC1 antibody binds to the C-terminal domain of NPC1 protein, we could not detect the truncated proteins that would be translated from the alleles with frameshift mutation. The apparent molecular mass of total NPC1 was 180 kDa, and of β-actin, it was 43 kDa.

### 2.8. Endoglycosidase H Assay

To remove immature N-linked glycans from NPC1, 20 µg of non-denatured protein samples were treated with recombinant endoglycosidase H (New England Biolabs, Frankfurt am Main, Germany) according to the manufacturer’s instructions with minor changes. In brief, cells were incubated with denaturing buffer (1 µL) at 37 °C for 10 min. The reaction was subsequently treated with Glycobuffer (2 µL), ddH20 (7 µL), and Endo H (1000U, 1 µL), and incubated at 37 °C for 2 h prior to analysis by Western blot as described above. The apparent molecular weight of the observed glycoforms was 170 kDa (Endo H-resistant NPC1) and 130 kDa (Endo H-sensitive NPC1), respectively.

### 2.9. Statistical Analyses

Data are presented as mean ± SD of at least three independent replicates. Statistical analysis was performed using the software GraphPad Prism 8 (GraphPad Software Inc., San Diego, CA, USA). The Shapiro–Wilk normality test was used to test the data sets for normal distribution. Statistical significance was assessed by ANOVA (for comparison of more than two means), followed by the Dunnett’s post hoc test or unpaired Student’s *t*-test (for comparison of two means). In cases where normality of the data distribution was not confirmed, the Kruskal–Wallis test was used in place of ANOVA, followed by Dunn’s post hoc test. Statistics with *p*-values < 0.05 were considered to indicate significant differences, with * = *p* < 0.05, ** = *p* < 0.01, and *** = *p* < 0.001.

## 3. Results

The overall aim of our study was to identify pharmacological chaperones used as potential therapeutic options for NP-C1. Therefore, we combined a high-throughput molecular docking with cell-based in vitro assays to elucidate pharmacological chaperones ameliorating key pathophysiological features of NP-C1 such as cholesterol accumulation, NPC1 protein expression, and localization. To fasten potential clinical translation of identified PCs, we used a drug bank of FDA-approved drugs for molecular docking. A total of three NPC1-deficient cell lines were investigated in primary fibroblasts, as well as their iPSC-derived neural and hepatic derivatives. Two patients were homozygous for different missense mutations (I1061T or Y394H) and one patient harbored a compound heterozygous mutation (E612D/P543Rfs*20). 

### 3.1. High Throughput in Silico Screening to Identify Potential Pharmacological Chaperones for NP-C1

**Figure 1:**
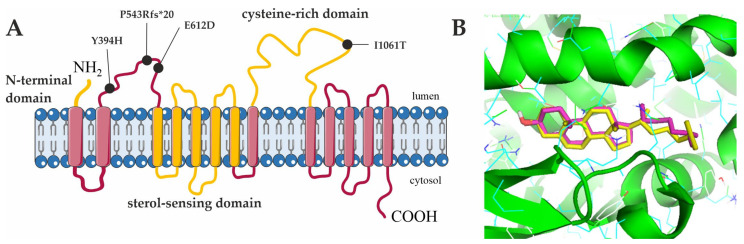
Scheme of the docking experiment. (**A**) Schematic representation of the NPC1 protein, showing three important domains in yellow: N-terminal domain, sterol-sensing domain, and cysteine-rich domain. The approximate locations of the NPC1 variants used in this study are indicated. (**B**) The comparison of the docked cholesterol (magenta) and the cholesterol from the crystal complex (yellow) revealed a similar binding pose, demonstrating that the docking simulation generates a reliable result.

In the first step, we performed a high throughput in silico screening to identify drugs with a high potential to bind to the N-terminal domain (NTD) of NPC1, positioned on the luminal side of the lysosome (Figure 1A). Here, we used FDA-approved drugs of the DrugBank using the Autodock vina software for docking simulation. The NTD structure of NPC1 in complex with cholesterol (PDB: 3GKI) was used as the receptor. The NTD of NPC1 protein is known as the binding site of the natural ligand cholesterol, as well as of its oxygenated derivatives such as 25-HC [17]. Specific ligand binding sites are often located at the interfaces between protein domains or subdomains [18]. In the case of NPC1, it has been shown that the NTD shares interfaces with the middle luminal domain and the cysteine-rich loop [19]. Therefore, ligands to the NTD are particularly suitable to stabilize the structure of the whole protein. To demonstrate that the docking procedure generates a reliable result, we performed a re-docking of cholesterol into the apo NPC1 structure of the cholesterol/NPC1 complex crystal structure (PDB: 3GKI) (Figure 1B). The docking pose of cholesterol in the re-docked structure (magenta) is similar to the binding pose of cholesterol in the cholesterol/NPC1 complex crystal structure (yellow). This result supports the accuracy of the docking procedure.

The docking results were analyzed with the Raccoon software and a list of the docking compounds with the binding score and binding efficiency was generated. Cholesterol, as the natural ligand of NPC1 protein, was used as a reference. Thus, all compounds with binding energy similar to the NTD or greater than that of cholesterol were scored as hit compounds (see Table 1). For subsequent in vitro assays, we used 25-HC as a reference as it has already been shown to act as a pharmacological chaperone in NPC1-deficient fibroblasts [20].

In the next step, we analyzed the cellular pathophysiological features of NP-C1 in patient-specific fibroblasts, as well as in cell types of the clinically most affected organs, namely, neural differentiated cells (NDCs) and hepatocyte-like cells (HLCs) derived from patient-specific iPSCs.

### 3.2. NPC1 Mutant Fibroblasts, Neural Differentiated Cells and Hepatocyte-like Cells Show Severe Cholesterol Accumulation

**Figure 2:**
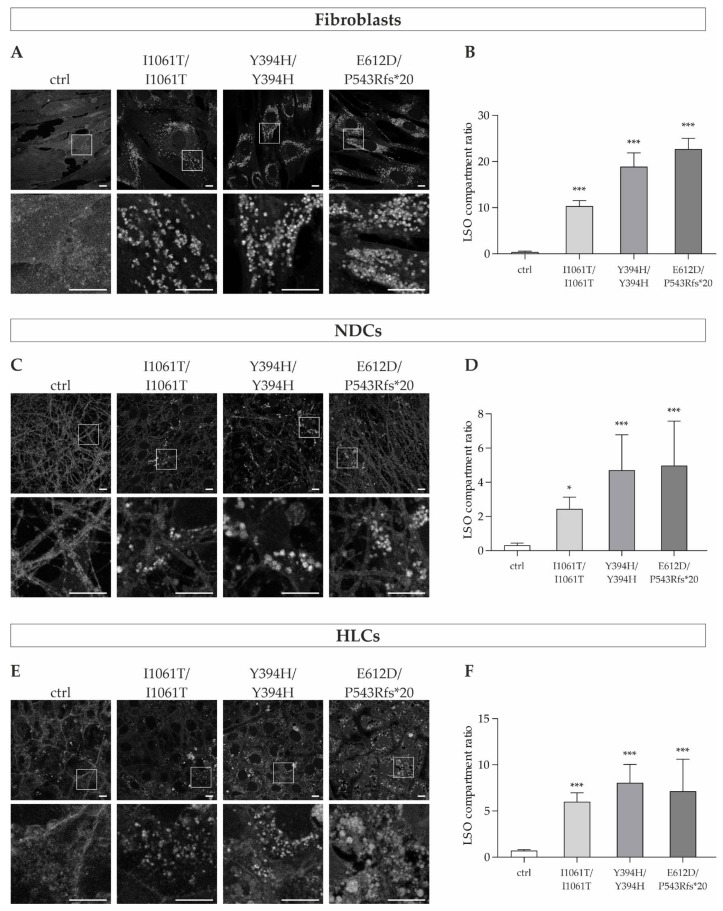
Cholesterol levels in NP-C1 patients’ fibroblasts, neural differentiated and hepatocyte-like cells. (**A**,**C**,**E**) Representative images of filipin-stained fibroblasts, neural differentiated cells and hepatocyte-like cells, respectively. Magnified images of the squared region of each image are shown below each one. In NPC1-deficient cell lines, bright fluorescent puncta are present, indicative of cholesterol accumulations in the lysosomes of the cells. In contrast, the control cells showed a weak overall fluorescence intensity with no fluorescent vesicles present. Scale bar = 10 µm. (**B**,**D**,**F**) Quantification of the lysosome-like storage organelles compartment ratio (LSO compartment ratio), which relates the cholesterol accumulation seen as fluorescent puncta to the total area of the cells. The LSO compartment ratio was significantly increased in fibroblasts, neural differentiated cells, and hepatocyte-like cells of NP-C1 patients compared to the control cell line, demonstrating an abnormal, increased amount of cholesterol in the cells. *N* = 8. * = *p* < 0.05, *** = *p* < 0.001 by one-way ANOVA followed by the Dunnett’s post hoc test compared to ctrl. NDCs = neural differentiated cells; HLCs = hepatocyte-like cells.

Since cholesterol accumulation is described as the hallmark of Niemann-Pick type C1 disease, we first examined the intracellular cholesterol content in the three cell types (fibroblasts, NDCs, and HLCs) of all patient-specific cell lines. Therefore, we performed filipin staining, which specifically detects free cholesterol in cells, to visualize and qualitatively assess the level of cholesterol accumulation (Figure 2A,C,E). Subsequently, the LSO compartment ratio (LSO, lysosome-like storage organelle) was determined for quantitative analysis of the cholesterol content. This relates the cholesterol accumulation in the perinuclear region to the total area of the cells [16] (Figure 2B,D,F).

In the fibroblasts of NPC1-patients, we observed a fluorescent pattern in the perinuclear region that was not present in the control cell line (Figure 2A). These fluorescent punctate vesicles are consistent with the accumulation of free cholesterol that is known to be located in late endosomes/lysosomes of the cells [21]. 

Analysis of the LSO compartment ratio (Figure 2B) revealed significantly increased levels in all NPC1-deficient cell lines compared to the control cell line, indicative of increased intracellular free cholesterol content. 

Similar to the observations in the patients’ fibroblasts, we could also observe a punctate pattern of bright staining in the iPSC-derived neural differentiated cells of NPC1-patients. These were not only present in the perinuclear region of the cell bodies, but also in their extensions (Figure 2C). In contrast, the control cell line showed a very low overall fluorescence intensity and no fluorescent vesicles, neither in the cell bodies nor in their extensions. The quantification of the fluorescence intensity of filipin by means of the LSO compartment ratio demonstrated a significantly increased amount of cholesterol in the mutant cell lines (Figure 2D).

In the hepatocyte-like cells, a similar tendency to that in the fibroblasts and neural differentiated cells was observed. The fluorescent puncta in the NPC1-deficient cell lines were primarily localized around the nucleus, as it was observed in the fibroblasts (Figure 2E). The mutant cell lines showed significantly increased LSO compartment ratios compared to the control cell line (Figure 2F). 

### 3.3. NPC1 Mutant Protein Is Expressed at Reduced Levels in Patient-Derived Cells and Is Retained in the Endoplasmic Reticulum

**Figure 3:**
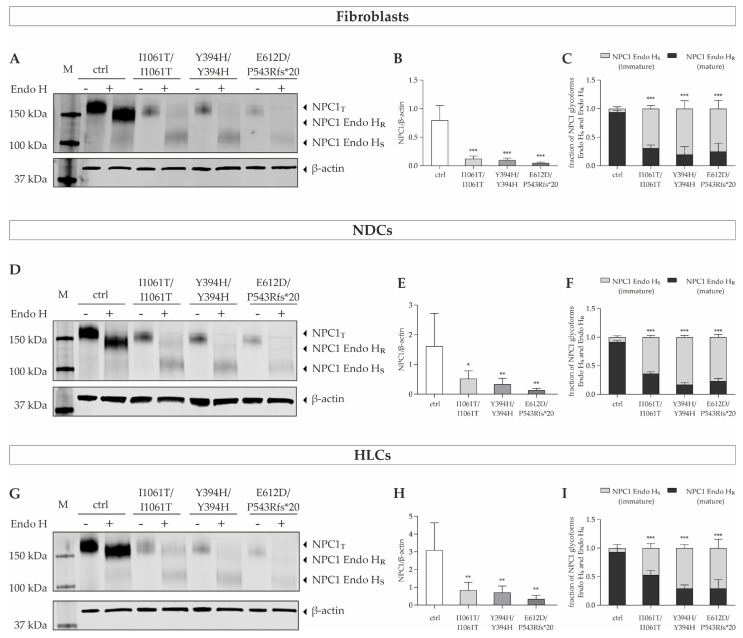
NPC1 expression in patients’ fibroblasts, neural differentiated and hepatocyte-like cells. (**A**,**D**,**G**) Western Blot analysis was performed to determine total NPC1 protein and maturation of NPC1 protein by digestion of the samples with endoglycosidase H in primary fibroblasts, NDCs, and HLCs, respectively. The fluorescence intensity of NPC1 band signals was normalized against the fluorescence intensity of β-actin band signals, which was used as a reference protein. (**B**,**E**,**H**) Quantification of the NPC1 band signals observed from the undigested samples demonstrated a significantly reduced amount of total NPC1 protein in all NPC1-deficient cell lines in comparison to the control cell line. *N* = 4–6. (**C**,**F**,**I**) Determination of the NPC1 glycoforms in patients’ fibroblasts, NDCs, and HLCs, respectively. The Endo H-resistant and Endo H-sensitive glycoforms of NPC1 protein are indicated as a fraction of total NPC1 (Endo H_R_ + Endo H_S_). Control cells predominantly show Endo H-resistant (Endo H_R_) NPC1 protein, while mutant NPC1 protein is mainly present in Endo H-sensitive (Endo H_s_) form, indicating retention in the ER. *N* = 6. * = *p* < 0.05, ** = *p* < 0.01, *** = *p* < 0.001 by one-way ANOVA, followed by the Dunnett’s post hoc test compared to ctrl. NDCs = neural differentiated cells; HLCs = hepatocyte-like cells; NPC1_T_ = total NPC1 protein; Endo H = Endoglycosidase H; Endo H_R_ = Endo H-resistant; Endo H_S_ = Endo H-sensitive; M = size marker.

To analyze the effect of the mutations on the NPC1 protein expression, we performed Western blot analysis in cells from the three NP-C1 patients compared to the control cell line. Figure 2 shows representative images of the observed NPC1 band pattern in patients’ fibroblasts, neural differentiated, and hepatocyte-like cells, as well as the corresponding quantification of the band signals. The total NPC1 protein was present as a distinct band located at ~180 kDa in the patient-specific fibroblasts (Figure 3A). Similar band patterns were observed in neural differentiated cells (Figure 3D) and hepatocyte-like cells (Figure 3G), indicating no major differences in neural and hepatic NPC1 protein. As expected, quantification demonstrated a reduced signal of total NPC1 protein in the NPC1-deficient fibroblast cell lines compared to normal control (Figure 3B), indicating that the mutations in the *NPC1* gene reduced the steady-state levels of the endogenously expressed NPC1 protein. Comparable to the patients’ fibroblasts, reduced NPC1 protein levels in comparison to the corresponding control cells were also seen in the NPC1-deficient iPSC-derived neural differentiated and hepatocyte-like cells (Figure 3E,H).

To draw conclusions about the intracellular localization of the NPC1 protein, we used a biochemical approach to analyze NPC1 trafficking. During translocation into the ER, the human NPC1 protein receives 14 N-linked glycans co-translationally [22]. The NPC1 trafficking can be monitored by treating cell lysates with endoglycosidase H (Endo H). Endo H is an enzyme that removes high mannose and hybrid type N-linked glycans from proteins in the ER but cannot cleave them after N-linked glycans are modified in the medial Golgi. Therefore, resistance to Endo H digestion indicates that the glycoprotein has trafficked beyond the ER in the secretory pathway, which is seen as a slow migrating NPC1 species on SDS-PAGE. In contrast, NPC1 protein that does not reach the medial Golgi is sensitive to Endo H and can be seen as a fast-migrating species on SDS-PAGE.

In control fibroblasts, NPC1 protein was almost completely present as the Endo H-resistant (Endo H_R_), slow migrating species (Figure 2C), indicating that the protein was efficiently transported out of the ER. In contrast, NPC1 mutant proteins were predominantly present as an Endo H-sensitive (Endo H_S_), more rapidly migrating species, while the Endo H-resistant species was markedly reduced (Figure 3C). This is in accordance with previous reports showing that more than 80% of NPC1^I1061T^ exhibits Endo H sensitivity [23], consistent with the notion that the mutant protein is retained in the ER prior to its degradation, hypothesizing that it is not located at its final destination, the lysosomal membrane. Similar to the patient-specific fibroblasts, the NPC1-deficient neural differentiated cells and hepatocyte-like cells also showed a decreased fraction of Endo H_R_ NPC1 species (Figure 3F,I), demonstrating a trafficking defect and retention in the ER. 

**Figure 4:**
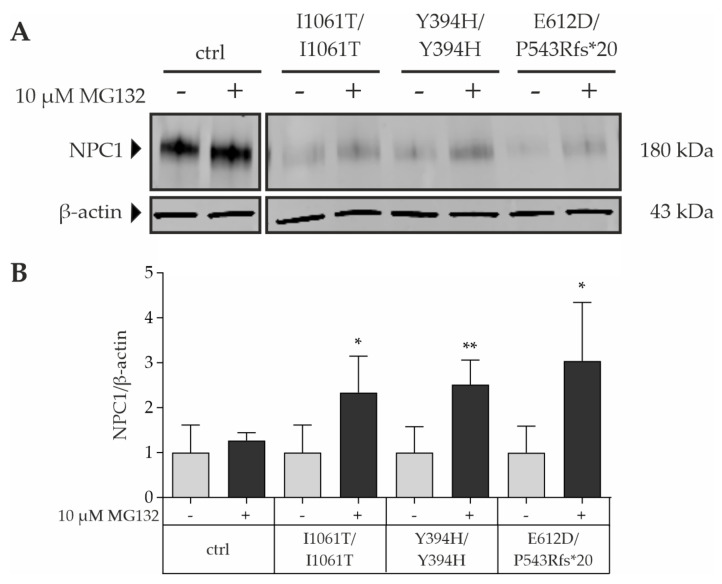
Proteasome inhibitor MG132 leads to an increase of total NPC1 mutant protein. (**A**) Representative Western blot analysis of NPC1 protein level in normal and NPC1-deficient fibroblasts after treatment with 10 µM MG132 for 24 h. β-actin was used as a reference protein. (**B**) Determination of the NPC1 band signals revealed a significant increase of mutant NPC1 protein upon treatment with 10 µM MG132 for 24 h, while the wild-type NPC1 protein did not change. This indicates that the mutant proteins are subjected to proteasomal degradation. *N* = 4; * = *p* < 0.05, ** = *p* < 0.01 by unpaired Student’s *t*-test compared to untreated samples.

Taken together, we could show a reduced expression and maturation of mutant NPC1 protein in all cell lines analyzed in this study, as well as in the three cell-type fibroblasts, NDCs, and HLCs. It has been well established that misfolded NPC1^I1061T^ protein is retained in the ER and targeted for ER-associated degradation (ERAD) [8]. To determine whether the reduction of NPC1 mutant protein that was seen in Western blot analysis (Figure 3B,E,H) is due to an increased rate of proteasomal degradation, control and NPC1-deficient fibroblast cell lines were treated with the proteasome inhibitor MG132 for 24 h and NPC1 protein levels were determined by Western blot (Figure 4). While treatment with MG132 had no effect on the NPC1 protein level of control cells, it led to a significant 2- to 3-fold increase of NPC1 protein in NPC1-deficient fibroblasts (Figure 4). These data are consistent with prior reports, indicating that NPC1 missense mutants, including I1061T, are rapidly degraded by the proteasome [8,24]. 

### 3.4. NPC1 Mutant Protein Displays Incorrect Intracellular Localization in Patient-Derived Cells

**Figure 5:**
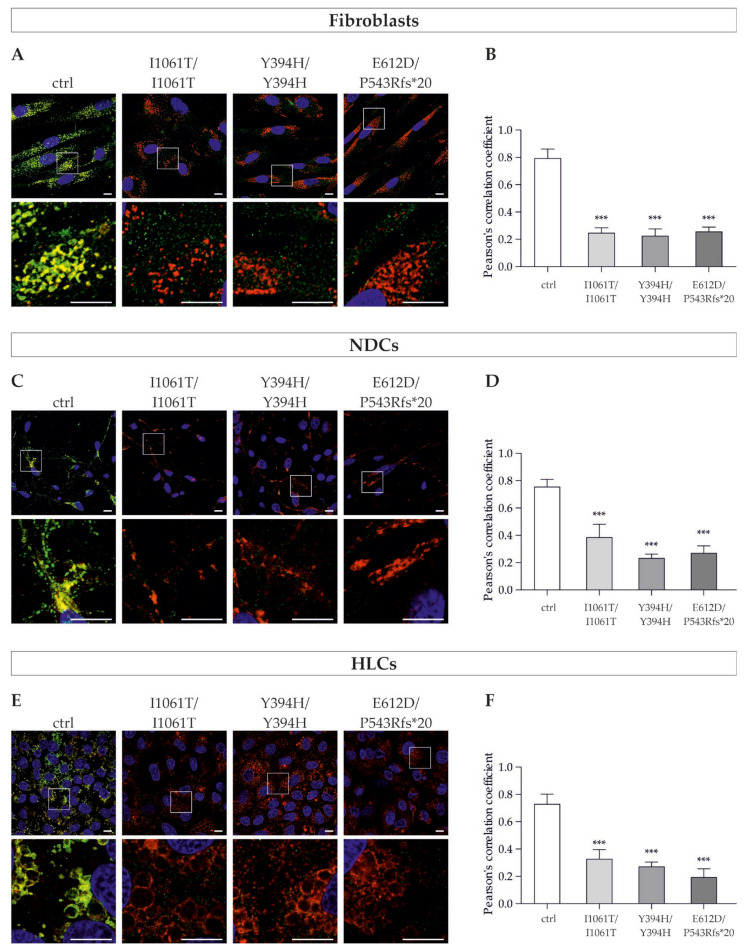
Intracellular localization of NPC1 protein in patients’ fibroblasts, neural differentiated, and hepatocyte-like cells. (**A**,**C**,**E**) Representative confocal images of immunofluorescence staining for NPC1 (green) and lysosomal marker LAMP2 (red) in fibroblasts, neural differentiated cells, and hepatocyte-like cells, respectively. DAPI was used to counterstain nuclei (blue). Magnified images of the squared region of each image are shown below each one. In cells of the control cell line, NPC1 was strongly expressed as seen by cytoplasmic puncta, which appear to strongly colocalize with LAMP2. In contrast, the overall fluorescent NPC1 signal intensity is very low in NPC1-deficient cells and visible colocalization with LAMP2 could not be observed, indicating massively reduced levels of NPC1 in the lysosomes of the cells. Scale bar = 10 µm. (**B**,**D**,**F**) Quantification of Pearson’s correlation coefficient revealed a weak NPC1/LAMP2 colocalization in all cell types of the NPC1-deficient cell lines, demonstrating a reduced amount of NPC1 protein in the lysosomes. *N* = 8. *** = *p* < 0.001 by one-way ANOVA, followed by the Dunnett’s post hoc test compared to ctrl. NDCs = neural differentiated cells; HLCs = hepatocyte-like cells.

The analysis of NPC1 glycoforms after Endo H digestion already provided information about the maturation of the NPC1 protein, revealing retention of mutant NPC1 protein in the ER while the wild-type protein is properly folded, trafficked through the secretory pathway, and probably localized to the lysosomes. To test this hypothesis, we visualized the NPC1 protein by immunofluorescence and examined its colocalization with the lysosomal marker LAMP2 by means of confocal microscopy (Figure 5A,C,E). The degree of colocalization between NPC1 and LAMP2 was quantified by calculating Pearson’s correlation coefficient (PCC) (Figure 5B,D,F). 

In the fibroblasts of the control cell line, the NPC1 protein was visible as cytoplasmic puncta that showed a strong colocalization with LAMP2 (Figure 5A). In contrast, mutant NPC1 fibroblasts showed a weak and diffused staining pattern that did not seem to colocalize with LAMP2 (Figure 5A). The quantitative analysis of NPC1/LAMP2 colocalization confirmed the visual impression (Figure 5B). The control cell line showed a high PCC value, demonstrating a strong colocalization, meaning that the majority of the NPC1 protein is localized in the lysosomes of the cells. In contrast, the PCC of the NPC1-deficient cell lines was significantly reduced, showing that there is less NPC1 protein in the lysosomal compartments. 

Similar to the observations in the patients’ fibroblasts, visually, a strong overlap of the fluorescence signals of NPC1 and LAMP2 was observed in the NDCs and HLCs of the control cell line, while the NPC1 fluorescence signal in the NPC1-deficient cell lines was very weak and showed a diffuse distribution pattern (Figure 5C,E).

Again, the PCC levels of the NPC1-deficient NDCs, as well as HLCs, were decreased in comparison to the control cell line.

In summary, we demonstrated several cellular pathological features of NP-C1 in fibroblasts, NDCs, and HLCs. We observed cholesterol accumulation in all NPC1-deficient cell lines, as well as reduced NPC1 protein levels and defective maturation and subcellular localization of the mutant protein. 

### 3.5. In Vitro Assessment of Identified Hit Compounds

In the next step, we analyzed the ability of the ten hit compounds that exhibited similar or higher binding affinity to the NTD of NPC1 protein than cholesterol (Table 1), to attenuate the cellular pathophysiological features of NP-C1 described above. To this end, we used patient-specific fibroblasts carrying two copies of the I1061T allele, since this mutant has been shown to be responsive to pharmacological chaperones [20].

**Figure 6:**
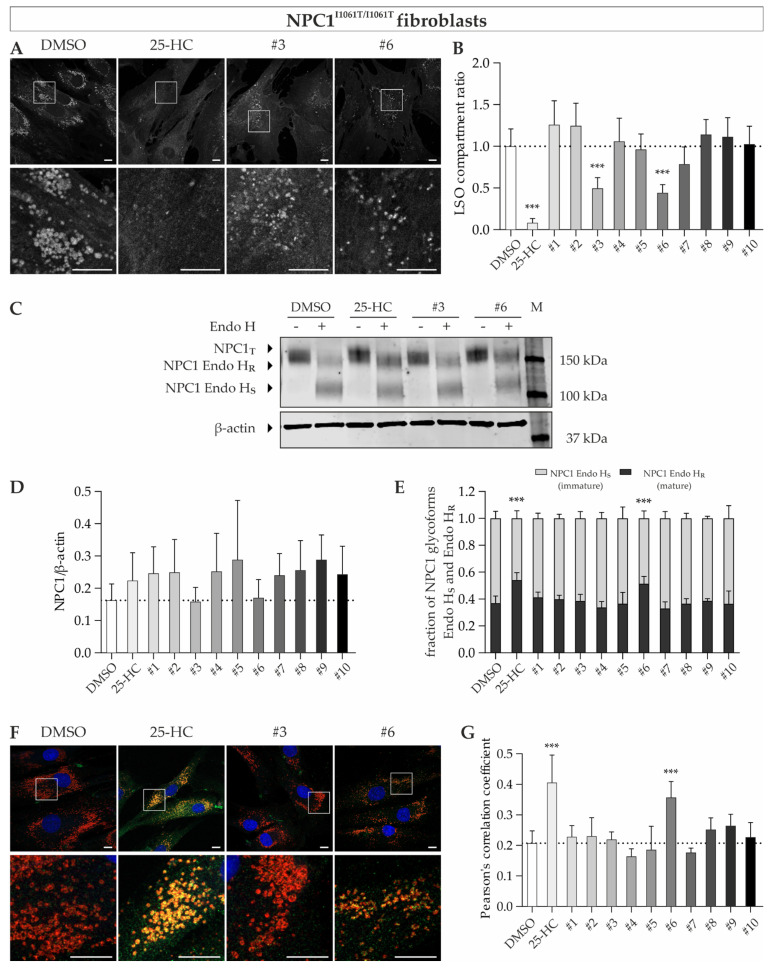
Treatment of NPC1^I1061T/I1061T^ fibroblasts with potential pharmacological chaperones. (**A**) Representative images of filipin-stained NPC1^I1061T/I1061T^ fibroblasts. (**B**) Quantification of LSO compartment ratio reveals decreased cholesterol content upon treatment with 25-HC, quinestrol (#3), and abiraterone acetate (#6). *N* = 8. (**C**) Representative image of Western blot analysis to determine total NPC1 protein (NPC1_T_) and post-ER glycoform of NPC1 protein (Endo H_R_). (**D**) Treatment with compounds did not lead to a change of NPC1_T_ levels. *N* = 4–11. (**E**) The Endo H-resistant and Endo H-sensitive glycoforms of NPC1 protein are indicated as a fraction of total NPC1 (Endo H_R_ + Endo H_S_). Determination of NPC1 glycoforms revealed an increase of Endo H_R_ portion upon treatment with 25-HC or abiraterone acetate (#6), indicating an ameliorated folding of NPC1 protein. *N* = 4–8. (**F**) Confocal images of immunofluorescence staining for NPC1 (green) and LAMP2 (red) in fibroblasts. DAPI was used to counterstain nuclei (blue). (**G**) Treatment with 25-HC or abiraterone acetate (#6) leads to a significant increase of NPC1 in lysosomes, as shown by increased PCC. *N* = 8. Scale bar = 10 µm. *** = *p* < 0.001 by one-way ANOVA, followed by Dunnett’s post hoc test compared to DMSO vehicle control. NPC1_T_ = total NPC1 protein; Endo H = Endoglycosidase H; Endo H_R_ = Endo H-resistant; Endo H_S_ = Endo H-sensitive; M = size marker.

First, we analyzed cholesterol accumulation by means of filipin staining and calculated the LSO compartment ratio to quantify the cholesterol content. The treatment with 10 µM 25-HC, quinestrol (#3) and abiraterone acetate (#6), respectively, led to a reduction of free cholesterol in fibroblasts^I1061T/I1061T^, as can be seen by a decreased amount and smaller and less fluorescent vesicles (Figure 6A). The quantification of the LSO compartment ratio supported the visual impression and revealed significantly reduced values upon treatment with 10 µM 25-HC, quinestrol (#3) and abiraterone acetate (#6), demonstrating diminished storage of free cholesterol by up to 92%.

Next, we determined whether the effect on cholesterol accumulation was due to increased levels of total NPC1 protein and/ or improved maturation of NPC1^I1061T^ protein. Western blot analysis revealed an increase of the Endo-H resistant portion of NPC1^I1061T^ protein upon treatment with 25-HC and abiraterone acetate (#6), indicating that these compounds promote maturation of mutant NPC1^I1061T^ protein (Figure 6E). However, total NPC1 protein levels did not change (Figure 6D). 

To assess whether these observations were accompanied by normal intracellular localization of the NPC1^I1061T^ protein in the lysosomal compartments, we performed immunofluorescence stainings of NPC1 and LAMP2 and subsequently performed colocalization analysis. In accordance with the findings in Western blot analysis, we observed increased LAMP2/NPC1 colocalization following the treatment with 25-HC and abiraterone acetate (Figure 6F,G). Interestingly, we did not observe an increase of Endo H-resistant NPC1 protein or increased LAMP2/NPC1 colocalization upon treatment with 10 µM quinestrol, even though the cholesterol accumulation was diminished by 50%.

### 3.6. Pharmacological Chaperones Are Effective in Different Mutants with NPC1 Missense Mutation 

**Figure 7:**
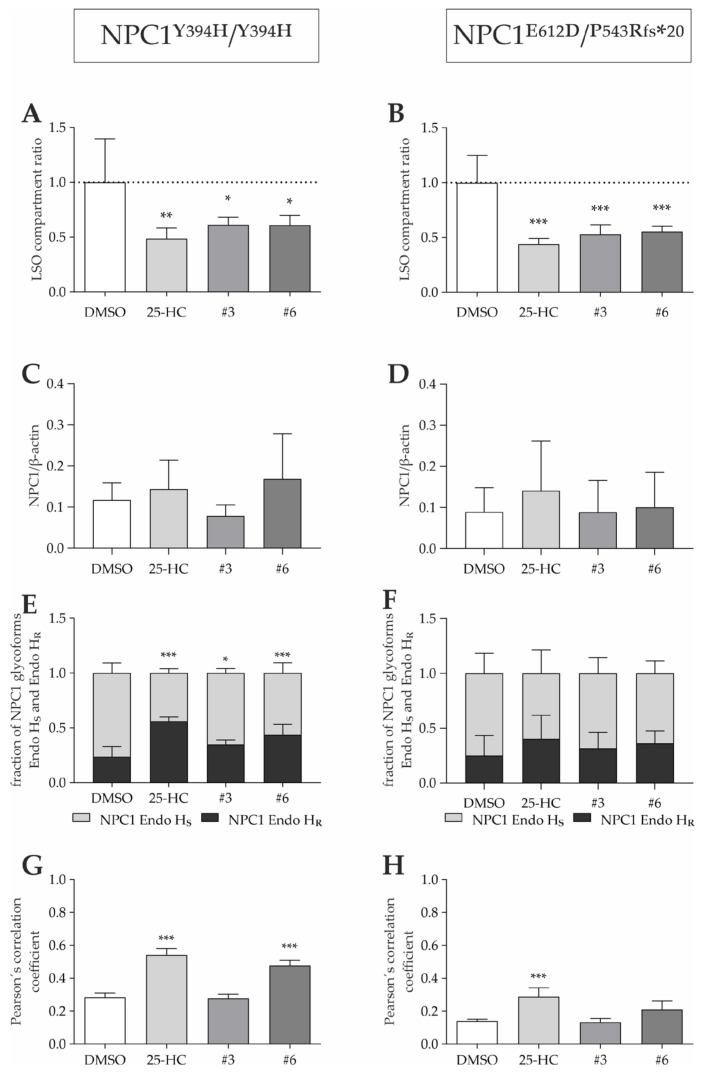
Treatment of NPC1-deficient fibroblasts with potential PCs. (**A**,**B**) Treatment with 10 µM 25-HC, quinestrol (#3), or abiraterone acetate (#6) for 48 h leads to a significant decrease in cholesterol accumulation. *N* = 5. (**C**,**D**) No change in total NPC1 protein levels was observed by Western blot. *N* = 4–7. (**E**,**F**) The Endo H-resistant and Endo H-sensitive glycoforms of NPC1 protein are indicated as a fraction of total NPC1 (Endo H_R_ + Endo H_S_). Treatment led to an increase in Endo H-resistant NPC1 protein in NPC1^Y394H/Y394H^ fibroblasts. *N* = 4–7. (**G**,**H**) Increased lysosomal NPC1 protein content was observed upon treatment with 25-HC and abiraterone acetate (#6) in NPC1^Y394H/Y394H^ fibroblasts, and upon treatment with 25-HC only in NPC1^E612D/P543Rfs*20^ fibroblasts. *N* = 4. * = *p* < 0.05, ** = *p* < 0.01, *** = *p* < 0.001 by one-way ANOVA, followed by the Dunnett’s post hoc test (in **A**,**B**,**E**–**H**) or Kruskal–Wallis test, followed by Dunn’s post hoc test (in **C**,**D**) compared to DMSO vehicle control. Endo H_R_ = Endoglycosidase H-resistant; Endo H_S_ = Endoglycosidase H-sensitive.

The applicability of pharmacological chaperones for NP-C1 is limited to missense mutations that retain residual function. As most of the NP-C1 disease-causing mutations identified so far are missense mutations, this may be the case for a large group of patients, however, this must be tested individually. Thus, we were interested in whether the hit compounds confirmed in NPC1^I1061T/I1061T^ fibroblasts, namely, abiraterone acetate and quinestrol, are also efficient in NPC1^Y394H/Y394H^ and NPC1^E612D/P543Rfs*20^ fibroblasts (Figure 7). 

Similar to the effect observed in NPC1^I1061T/I1061T^ fibroblasts, the treatment with 25-HC, quinestrol, and abiraterone acetate led to an amelioration of cholesterol accumulation in NPC1^Y394H/Y394H^ and NPC1^E612D/P543Rfs*20^ fibroblasts (Figure 7A,B). Western blot analysis partly confirmed these results as the treatment with 25-HC, quinestrol, or abiraterone acetate also led to a significant increase of the post-ER form (Endo H_R_) of NPC1^Y394H^ protein in fibroblasts (Figure 7E), while the total NPC1 protein remained unchanged (Figure 7C). The same tendency could be observed in NPC1^E612D/P543Rfs*20^ fibroblasts, even though the observed differences failed to meet statistical significance (Figure 7F,D). Furthermore, the treatment with 25-HC or abiraterone acetate improved the delivery of the NPC1^Y394H^ mutant protein to the lysosomal compartment as shown by an increased Pearson’s correlation coefficient (Figure 7G). The same could be shown upon 25-HC treatment in NPC1^E612D/P543Rfs*20^ fibroblasts (Figure 7H). 

Taken together, we could show that, besides the NPC1^I1061T^ mutant protein, other NPC1 mutant variants such as NPC1^Y394H^ and NPC1^E612D^ are subjected to an increased rate of proteasomal degradation (Figure 4) that can, in part, be rescued by pharmacological chaperones (Figure 7). As most NPC1 variants are missense mutations, this shows that pharmacological chaperones can be beneficial for a large group of NP-C1 patients.

Finally, we were interested in the effect of quinestrol and abiraterone acetate in cell types, which are severely affected in NP-C1. 

### 3.7. Treatment in Disease-Affected Cell Types Neural Differentiated Cells and Hepatocyte-like Cells

**Figure 8:**
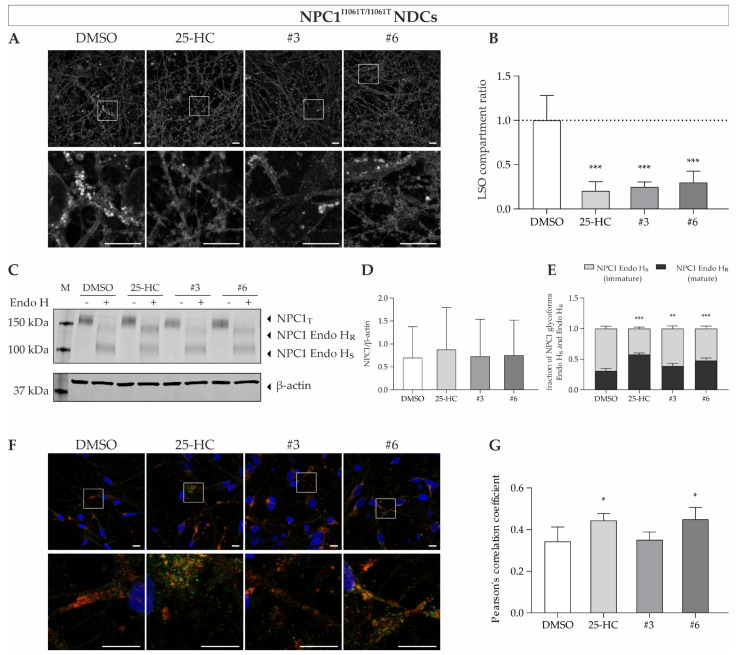
Treatment of NPC1^I1061T/I1061T^ NDCs with potential pharmacological chaperones. (**A**) Representative images of filipin-stained NPC1^I1061T/I1061T^ NDCs. Intensive fluorescent puncta indicative of cholesterol accumulation are decreased upon treatment with the substances. (**B**) Quantification of the LSO compartment ratio revealed a significant decrease in cholesterol accumulation based on the filipin staining in NDCs. *N* = 8. (**C**) Representative Western blot of NDC protein samples. Protein lysates were digested with endoglycosidase H for the detection of post-ER glycoform of NPC1 (Endo H_R_) that was visibly increased upon treatment with the compounds. (**D**) Quantification revealed no change of total NPC1 protein. *N* = 6–7. (**E**) The Endo H-resistant and Endo H-sensitive glycoforms of NPC1 protein are indicated as a fraction of total NPC1 (Endo H_R_ + Endo H_S_). Determination of NPC1 glycoforms demonstrates an increase in mature, Endo H-resistant NPC1 protein in 25-HC, quinestrol, or abiraterone acetate treated NDCs. *N* = 6–7. (**F**) NPC1 (green) and LAMP2 (red) were visualized in NDCs by means of confocal microscopy. DAPI was used to counterstain nuclei (blue). (**G**) An increase of Pearson’s correlation coefficient in 25-HC and abiraterone acetate treated NDCs indicates a larger portion of NPC1 protein in the lysosomal compartment compared to vehicle control. *N* = 4. Scale bar = 10 µm. * = *p* < 0.05, ** = *p* < 0.01, *** = *p* < 0.001 by one-way ANOVA, followed by the Dunnett’s post hoc test (in **B**,**E**,**G**) or the Kruskal–Wallis test, followed by Dunn’s post hoc test (in **D**) compared to DMSO vehicle control. NDCs = neural differentiated cells; NPC1_T_ = total NPC1 protein; Endo H = Endoglycosidase H; Endo H_R_ = Endo H-resistant; Endo H_S_ = Endo H-sensitive; M = size marker.

**Figure 9:**
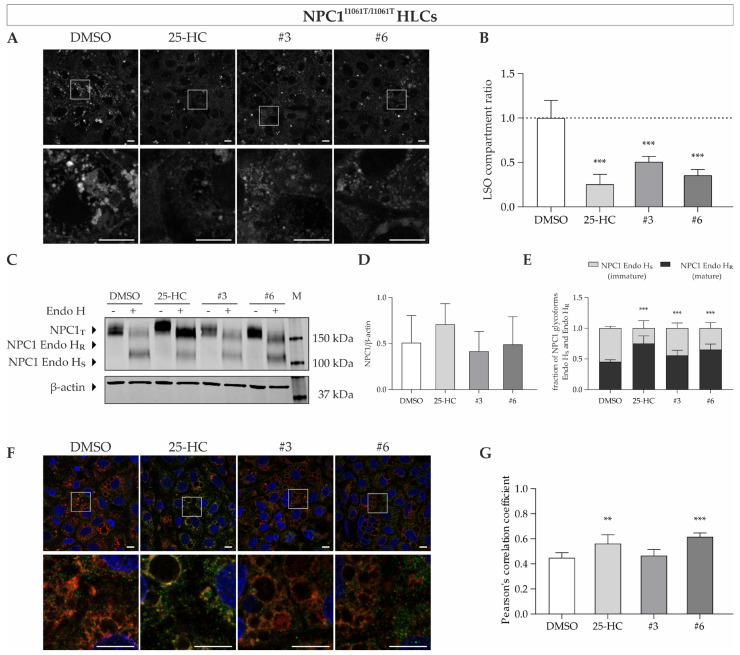
Treatment of NPC1^I1061T/I1061T^ HLCs with potential pharmacological chaperones. (**A**,**B**) Filipin staining and its quantitative analysis by means of LSO compartment ratio revealed a significant decrease in the HLCs cholesterol content upon treatment with the substances. *N* = 5. (**C**) Western blot analysis was performed to determine the effect of the substances on the total NPC1 protein levels and maturation of NPC1 protein as represented by Endo H-resistance. (**D**) While total NPC1 protein expression did not significantly change (*N* = 6), (**E**) mutant NPC1^I1061T^ protein in HLCs has been shown to fold more properly through the action of 25-HC, quinestrol, or abiraterone acetate as shown by increased levels of mature NPC1 protein (Endo H_R_). The Endo H-resistant and Endo H-sensitive glycoforms of NPC1 protein are indicated as a fraction of total NPC1 (Endo H_R_ + Endo H_S_). *N* = 4. (**F**,**G**) Immunofluorescence staining of NPC1 (green) and LAMP2 (red) in HLCs and its assessment by means of colocalization analysis demonstrates an increased lysosomal NPC1 protein content in 25-HC or abiraterone acetate treated HLCs. DAPI was used to counterstain nuclei (blue). *N* = 6. Scale bar = 10 µm. ** = *p* < 0.01, *** = *p* < 0.001 by one-way ANOVA, followed by the Dunnett’s post hoc test compared to DMSO vehicle control. HLCs = hepatocyte-like cells; NPC1_T_ = total NPC1 protein; Endo H = Endoglycosidase H; Endo H_R_ = Endo H-resistant; Endo H_S_ = Endo H-sensitive; M = size marker.

In the next step, we used NDCs and HLCs derived from induced pluripotent stem cells carrying the I1061T mutation, as NP-C1 patients suffer from severe neurological symptoms and often show pronounced hepatosplenomegaly [1]. The compounds quinestrol (#3) and abiraterone acetate (#6), which demonstrate an effect in NPC1^I1061T/I1061T^ fibroblasts, were used to assess the effect in NDCs and HLCs. Indeed, we could observe a significant decrease in cholesterol accumulation upon treatment with these substances in NDCs and HLCs (Figure 8A,B and Figure 9A,B). This was supposedly seen as a consequence of increased mature NP-C1 protein as expressed by the post-ER glycoform of NPC1 (Endo H_R_) (Figure 8C,D and Figure 9C,D), while the total NPC1 protein was unchanged (Figure 8E and Figure 9E). Interestingly, we could see an increase in this post-ER form upon treatment with quinestrol, although this failed to meet statistical significance in the NPC1-deficient fibroblasts (Figure 6E). The increase of NPC1 protein abundance was associated with increased localization of NPC1 protein within the lysosomal compartment, at least upon treatment with 25-HC and abiraterone acetate, as could be shown by colocalization analysis of NPC1 and LAMP2 (Figure 8F,G and Figure 9F,G). Similar to the results in NPC1^I1061T/I1061T^ fibroblasts, no increased lysosomal localization of mutant NPC1 was seen in neural differentiated and hepatocyte-like cells upon treatment with 10 µM quinestrol for 48 h (Figure 8F,G and Figure 9F,G).

Overall, we have shown that NPC1-deficient iPSC-derived NDCs and HLCs exhibit important pathophysiological features of NP-C1. Furthermore, we demonstrated that these cell types respond to treatment with pharmacological chaperones in a manner similar to patient-specific fibroblasts. Thus, these cells represent an excellent cell model to study cell-type-specific readouts and are a valuable addition to model systems for studies on the pathophysiological mechanisms of NP-C1.

## 4. Discussion

Since there is still no specific treatment for NP-C1, different screening campaigns are being conducted with the aim of identifying compounds from which treatment strategies for NP-C1 can be derived. Different strategies on how to treat NP-C1 have been pursued, as well as different strategies to identify potential drug candidates. In terms of treatment strategies, promising therapeutic approaches have aimed to reduce substrate accumulation found in NP-C1, such as treatment with cyclodextrins and miglustat. Cyclodextrins are cyclic oligosaccharides with a lipophilic cavity [25] that realize the egress of free cholesterol in an NPC1-independent manner [26]. 

Treatment with miglustat, an inhibitor of the glycosylceramide synthase, results in a reduction of glycosphingolipids. Furthermore, it has been shown to attenuate key neurological manifestations of NP-C1 patients [27,28,29] and consequently was approved in 2009 by the European Medicines Agency for the treatment of NP-C1. However, these approaches aim at a direct reduction of substrate loading and leave an effect on the function of the NPC1 protein out of sight. In fact, most of the disease-causing mutations in *NPC1* identified so far, including the most prominent I1061T, are missense mutations [30] associated with a folding defect and premature degradation rather than intrinsic loss of function [8]. Accordingly, efforts have been made to identify drug candidates that lead to upregulation of NPC1 variant expression, thereby increasing the NPC1 functional capacity. This resulted, among others, in the identification of ryanodine receptor antagonists [31], histone deacetylase inhibitors (HDACi, [32,33]) and arimoclomol [34,35] to modulate the NPC1 folding environment.

However, neither of these strategies directly target the NPC1 protein. Thus, there is a need for more specific therapeutics that address the underlying folding defect that is seen in most NP-C1 patients with a missense mutation. In this context, pharmacological chaperones that specifically bind to and stabilize the target protein can be used, thereby correcting its pathogenic misfolding, promoting its trafficking to the subcellular destination, and restoring its functional capability. The utility of pharmacological chaperones in the context of NP-C1 has been repeatedly explored using different approaches by the working group of Ohgane [20,36,37,38]. Oxysterols and their derivatives, such as 25-HC, were first identified to act as pharmacological chaperones for NP-C1 disease due to their conformational similarity to the natural ligand cholesterol [20,36]. To develop more stable and drug-like chaperones, the working group developed phenanthridine-6-one derivatives as non-steroidal pharmacological chaperones [37]. However, until now, this did not yield to clinical testing of PCs for treatment of NP-C1.

### 4.1. Efficient Screening by Pre-Selection of Potential Candidates through High Throughput in Silico-Screening

Our study was designed to identify novel compounds that act as pharmacological chaperones by using a combinatorial approach of high throughput in silico screening and in vitro cell-based assays.

We used a library of 2162 FDA-approved substances to allow the rapid translation from research to clinics. A comparable approach, known as drug-repurposing [39], was used by Shioi and colleagues, who screened 758 approved drugs [31] to identify potential chaperone drugs for NP-C1. In contrast to our study, they used an image-based screening method that monitors correction of mislocalization of the NPC1 protein and found a subset of azole antifungals, including itraconazole as the most promising hit compound. Another strategy based on image-based high-content screening was performed by Pugach and colleagues, wherein this screening was not solely focused on pharmacological chaperones [40]. A total of 3532 compounds, including 2013 FDA-approved drugs, were screened based on a dual readout on NPC1^I1061T/I1061T^ fibroblasts. Besides several HDACis, the antimicrobial compound alexidine dihydrochloride was found to reduce both cholesterol accumulation and LAMP1 signal [40]. In contrast to these studies, we used an in silico approach as a first step to reduce the number of compounds to be tested in vitro, which is time-consuming, expensive, and labor-intensive. The in silico molecular docking was based on the x-ray crystallographic structure of the N-terminal domain (NTD) of the NPC1 protein. We have chosen the NTD as it is described as the cholesterol-binding site [41]. This binding site does not only bind cholesterol but also its oxygenated derivatives 25-HC and 27-HC [17]. Interestingly, Ohgane and colleagues later showed that oxysterol-based chaperones exert their effect through sterol-binding site(s) other than the N-terminal domain (NTD) [20]. However, the exact location of the non-NTD sterol-binding site(s) is not known. In contrast, the three-dimensional structure of the NTD used in this study is well described and available in a high resolution of 1.8 Å [17], which allowed us to rationally identify ligands that potentially interact with the NPC1 protein and thus act as potential pharmacological chaperones. 

As a result of the molecular docking, ten potential pharmacological chaperones were identified. These potential candidates were subsequently tested in vitro, using patient-specific fibroblasts, carrying the most frequent *NPC1* mutation (I1061T) as a first step, followed by investigations to confirm the efficacy of these substances in disease-affected cell types such as neural differentiated cells and hepatocyte-like cells. To the best of the author’s knowledge, this is the first study using these disease-relevant cell types to investigate compounds that directly target the NPC1 protein, which increases the clinical relevance of the identified substances.

### 4.2. Discovery of Abiraterone Acetate and Quinestrol as Pharmacological Chaperones for NP-C1 Disease

The most potent compound found in this study was abiraterone acetate (Zytiga^®^), which is the ester prodrug of abiraterone. Following oral administration, abiraterone acetate is hydrolyzed to the active metabolite abiraterone that acts through inhibition of CYP17 as an antiandrogen and is approved in the European Union and the US, in combination with prednisone or prednisolone, for the treatment of men with metastatic castration-resistant prostate cancer [42]. Here, our data indicate that abiraterone acetate has a high binding affinity to the N-terminal domain of the NPC1 protein (Table 1) and affects multiple molecular processes in NPC1-deficient cells. 

First, analysis of filipin-stained cells revealed a significant decrease in LSO compartment ratio upon treatment with abiraterone acetate, indicating a decrease of cholesterol accumulation in all three cell types of NPC1^I1061T/I1061T^ cells (Figure 4B, Figure 6B, and Figure 7B). A diminished cholesterol accumulation in NPC1^I1061T/I1061T^ fibroblasts has also been shown upon treatment with compounds identified through other, image-based strategies, such as alexidine dihydrochloride [40] and itraconazole [38], albeit comparison of efficiency is hardly feasible due to different treatment conditions. In our case, decreased cholesterol levels were shown to be a consequence of increased mature NPC1 protein levels, rather than an increase of total NPC1 protein, probably through an enhancement of folding efficiency realized through the binding of abiraterone acetate to the NTD. In contrast, the hit compound alexidine dihydrochloride identified by Pugach and colleagues increased total NPC1 protein levels in both control and NPC1^I1061T/I1061T^ fibroblasts. Furthermore, treatment induced both mature and immature NPC1^I1061T^ protein glycoforms, indicating a more unspecific mode of action [40]. The hit compound that was identified by Shioi and colleagues, itraconazole, increased total NPC1 protein levels in a comparatively short treatment time of 21 h. However, this resulted in a band of higher electrophoretic mobility, probably due to an inhibitory effect on N-glycosylation, a process generally known to enhance protein stability [43].

In our study, we showed that increased mature NPC1 protein was accompanied by a stronger colocalization with the lysosomal marker LAMP2 (Figure 4G, Figure 6G and Figure 7G), indicating the correction of subcellular localization upon treatment with abiraterone acetate. Taken together, our data herein demonstrate that abiraterone acetate, a substance that has not been described before in the context of NP-C1, can correct the trafficking defect linked with NPC1 mutants and could, therefore, work as a potent pharmacological chaperone in NP-C1 patients. 

Furthermore, abiraterone acetate is likely to cross the blood–brain barrier, which is of special importance to treat the neurodegeneration in NP-C1. Until now, penetration across the blood–brain barrier by abiraterone acetate only has been shown in animal studies and has not been investigated in humans [44]. However, due to its small size of 391,5 Da, which defines abiraterone acetate as a small molecule [45], it is likely to pass the blood–brain barrier and yield brain concentrations that will impact the folding defect of neurodegenerative disease NP-C1.

Another potential pharmacological chaperone for NPC1 identified through our screening is quinestrol. Quinestrol is a synthetic estrogen, synthesized in 1961 [46], that was used in menopausal hormone therapy, hormonal birth control, and occasionally to treat breast and prostate cancer, but is not currently marketed in the United States [47]. 

Interestingly, quinestrol was also part of the study of Pugach and colleagues but was not identified as a hit compound in terms of reduction of cholesterol accumulation or LAMP1 signal [40]. This stays in contrast to our study that showed a significant reduction of cholesterol content in all three cell types carrying the I1061T mutation in homozygosity upon treatment with quinestrol (Figure 4B, Figure 6B, and Figure 7B). Of note, this was accompanied by a statistically significant correction of the NPC1^I1061T^ trafficking defect only in the NDCs and HLCs as shown by increased Endo H_R_ species (Figure 6E and Figure 7E), while in fibroblasts, quinestrol treatment did not provide a statistically significant increase of mature NPC1 protein (Figure 4E). Furthermore, we did not detect a difference in lysosomal localization of NPC1, as shown by unaltered NPC1/LAMP2 colocalization in all three cell types (Figure 4G, Figure 6G, and Figure 7G). This either suggests that even the smallest effects on I1061T trafficking can lead to a major reduction in cholesterol levels that was missed by Pugach and colleagues [40] and/or that the NPC1^I1061T^ is subjected to a different degradation process upon reaching the lysosomes. The latter suggestion was recently substantiated by Schultz and colleagues, suggesting FAM134B-dependent ER-phagy to be the primary degradation process in NPC1-mutants [30,48], which stays in contrast to previous studies that assumed that NPC1 is degraded primarily through the ubiquitin–proteasome system [8]. 

In summary, we showed a beneficial effect of abiraterone acetate and quinestrol on cholesterol accumulation. While previously proposed pharmacological chaperones, such as oxysterol-derivatives [20] and itraconazole [38], are thought to exert their effect through a binding site different than the NTD, we assume that the effect of quinestrol and abiraterone acetate is based on the strong interaction to the NTD, as predicted by in silico screening, and its subsequent stabilization of the misfolded protein.

### 4.3. Pharmacological Chaperones Display an Effective Treatment Strategy for a Large Group of NP-C1 Patients

Of note, PCs appear not to be efficient in certain NP-C1 variants. In fact, they are not useful in cases where the protein is completely absent because the gene is affected by a deletion, a stop gain mutation, or a splicing mutation [49]. Instead, the applicability of the PC approach is directed towards missense variants that retain some residual function and must be tested individually. 

While the mutant protein of NPC1^I1061T/I1061T^ fibroblasts has been shown to respond to therapeutics that correct its maturation and trafficking defect [20], other cell lines have not been subjected to pharmacological chaperone treatment yet. Thus, in addition to fibroblasts harboring I1061T mutation in homozygosity, we assessed the efficacy of abiraterone acetate and quinestrol in two other cell lines, carrying different mutations, namely NPC1^Y394H/Y394H^ and NPC1^E612D/P543Rfs*20^ fibroblasts. While the treatment with quinestrol and abiraterone acetate did lead to an increase in Endo H-resistant (mature) NPC1 protein in the NPC1^Y394H/Y394H^ fibroblasts, no significant increase was measured in the compound heterozygous cell line, even though a tendency was seen (Figure 5). We observed a comparable effect in the colocalization analysis, where abiraterone acetate increased lysosomal NPC1 localization in NPC1^Y394H/Y394H^ but not in the compound heterozygous cell line. This is probably due to the fact that in NPC1^E612D/P543Rfs*20^ fibroblasts, only one allele with missense mutation is applicable to the treatment with PCs, while in NPC1^Y394H/Y394H^ fibroblasts, both are. Independent of mutations, both quinestrol and abiraterone acetate resulted in significant decreases in cholesterol accumulation in both cell lines, suggesting that PCs represent a promising treatment strategy to delay disease onset and slow clinical progression for a larger subset of NP-C1 patients. 

### 4.4. Perspectives

In this study, we identified abiraterone acetate and quinestrol as potential pharmacological chaperones for the treatment of NP-C1. In contrast to other studies, we took advantage of a pre-selection of the compounds to be tested in vitro by performing an in silico molecular docking to identify potential candidates in advance. In subsequent in vitro evaluation, two of ten potential pharmacological chaperones were found to be effective in reducing cholesterol accumulation in NPC1^I1061T/I1061T^ fibroblasts. To ultimately prove that abiraterone acetate and quinestrol are acting as pharmacological chaperones and not through an unknown mechanism, the direct binding should be investigated in the future. To this end, photo-crosslinking studies have been proven to be effective in assessing the binding of a compound to the NPC1 protein [20,38]. Despite this, on the basis of the in silico results predicting strong interaction of abiraterone acetate and the NTD of the NPC1 protein, as well as the increased amount of mature, Endo H-resistant NPC1 protein and lysosomal localization, we assume abiraterone acetate to act as a pharmacological chaperone. However, further studies are needed to clarify the effect of quinestrol.

Another future direction could be the development of second-generation pharmacological chaperone [50]. First-generation pharmacological chaperones are defined as substances that bind to the active site, acting as competitive inhibitors. This concept has also been extended to ligands for transporters, such as Cystic Fibrosis Transmembrane Conductance Regulator (CFTR), and thus might also be applicable for the NPC1 protein. The compounds identified in this study are predicted to bind to the NTD of NPC1, depicting the cholesterol-binding site. Thus, it might appear paradoxically using these compounds to restore the function of the mutant protein. Indeed, it is a prerequisite for an effective pharmacological chaperone to relieve the binding pocket upon successful trafficking to the lysosomes. Alternatively, second-generation pharmacological chaperones can be used that act as non-inhibitory ligands, targeting previously unknown binding pockets. Recently, a second-generation corrector, tezacaftor, was developed for the treatment of cystic fibrosis based on the structure of a first-generation corrector, lumacaftor, demonstrating rescue of CFTR folding and function with better pharmacokinetic properties and fewer adverse effects [51]. Similar to this approach, the small molecule abiraterone acetate identified in this study could serve as a promising lead compound for further structural optimization to develop a second-generation pharmacological chaperone for NP-C1 that could be retested by the same cell-based methods used in this study.

In summary, in this study, we showed that in silico docking coupled with biochemical assays are capable of identifying interacting molecules for NPC1, thus displaying an effective screening approach. It is reasonable to expect that the most potent compound identified through this approach, namely abiraterone acetate, would be beneficial for a number of misfolding NPC1 mutations and thus displays a rational basis for a translation into a therapeutic approach of NP-C1.

## Figures and Tables

**Table 1:** The list of the top hit compounds obtained from in silico screening.

#	DrugBank ID	Compound Name	Binding Energy [kcal/mol]	Binding Efficiency [kcal/mol]
1.	DB00378	Dydrogesterone	−12.6	−0.548
2.	DB06710	Methyltestosterone	−11.6	−0.527
3.	DB04575	Quinestrol	−11.6	−0.43
4.	DB09280	Lumacaftor	−12.1	−0.367
5.	DB00984	Nandrolone phenpropionate	−11.6	−0.387
6.	DB05812	Abiraterone *	−11.4	−0.438
7.	DB06210	Eltrombopag	−11.4	−0.345
8.	DB00977	Ethinyl Estradiol	−11.3	−0.514
9.	DB01420	Testosterone propionate	−11.3	−0.452
10.	DB12598	Nafamostat	−11.3	−0.435
reference	DB04540	Cholesterol	−11.3	−0.404
reference	DB04705	25-HC	−11.7	−0.403

* The ester prodrug abiraterone acetate instead of abiraterone was used for in vitro analyses. Following oral administration, abiraterone acetate is hydrolyzed to the active metabolite abiraterone.

## Data Availability

The data presented in this study are available on request from the corresponding author.

## Abbreviations

25-HC	25-hydroxycholesterol
BSA	Bovine Serum Albumin
DB	DrugBank
DMSO	Dimethyl sulfoxide
Endo H	Endoglycosidase H
ER	Endoplasmic reticulum
ERAD	Endoplasmic reticulum-associated degradation
FDA	Food and Drug Administration
HLCs	Hepatocyte-like cells
iPSCs	Induced pluripotent stem cells
LDL	Low Density Lipoprotein
LSD	Lysosome-like storage organelle
LSM	Laser Scanning Microscope
LSO	Lysosome-like storage organelle
NDCs	Neural differentiated cells
NPC1	Niemann-Pick type C1 protein
NP-C1	Niemann-Pick disease type C1
NTD	N-terminal domain
PC	Pharmacological chaperone
PCC	Pearson’s correlation coefficient
PDB	Protein Data Bank
PFA	Paraformaldehyde
